# Resolving flow and mass transport in a healthy subject-specific aorta using large eddy simulation

**DOI:** 10.1186/1532-429X-14-S1-W60

**Published:** 2012-02-01

**Authors:** Jonas Lantz, Matts Karlsson

**Affiliations:** 1 Department of Management and Engineering, Linköping University, Linköping, Sweden

## Background

An increased level of low-density lipoprotein (LDL) has been shown to promote the accumulation of cholesterol within the intima layer of large arteries, which can be connected to the genesis of atherosclerosis. The LDL concentration on the endothelium has been found to be flow dependent, suggesting a relationship between the development of atherosclerosis and flow dynamics. In this study, we use a scale resolving turbulence model (Large eddy simulation, LES) to compute the flow and mass transfer inside a subject-specific human aorta, in order to investigate the relation between the flow and the LDL accumulation on the arterial wall.

## Methods

The Reynolds number is in the transitional regime and disturbed flow was found, which motivated the use of LES. The complete aorta and flow profiles in the ascending aorta were obtained by MRI measurements; 3D volume data was reconstructed to a resolution of 0.78x0.78x1.00 mm^3^, while time-resolved aortic flow profiles were reconstructed to 40 timeframes per cardiac cycle with a spatial resolution of 1.37x1.37 mm^2^. Aortic geometry from the MRI images was extracted with a 3D level set algorithm. The LES model used the WALE sub-grid model, which has been shown to accurately compute near-wall velocity and is able to handle transition. LDL was modeled as a passive scalar and transport into the arterial wall was handled with a filtration boundary condition following Wada and Karino (Biorheology 36, 207-223). Lagrangian particles were introduced to be to properly investigate the flow field using path lines.

## Results

Due to the disturbed nature of the flow, consecutive cardiac cycles were not identical. A phase average based on a large number of cardiac cycles was therefore computed, to get reliable statistical results from the LES simulation. In total, 50 cardiac cycles were simulated, yielding over 2.5 Billion surface values to post-process. The Lagrangian particle tracks revealed a helical flow motion in the aortic arch, similar to the flow described by Kilner et al. (Circulation, 88:2235-2247), and complex transitional flow was found during the latter part of systole, with disturbed flow in the arch and descending aorta. A large recirculation region formed during systole on the inner curvature of the descending aorta, with a fluctuating reattachment point during the cardiac cycle. The LDL concentration boundary layer was sensitive to near-wall flow features; regions with high near-wall velocities had a lower concentration level compared to regions where the flow was stagnant, and flow disturbances resulted in both temporal and spatial changes of the LDL level.

## Conclusions

In general, a low wall shear stress level corresponded to an elevated LDL level and vice versa, indicating an inverse relationship between LDL concentration and wall shear stress. Both near-wall velocity and wall shear stress are important to resolve when modeling the LDL concentration.

## Funding

Swedish research council VR 2010-4282 and computational resources grant No. SNIC022/09-11.

